# LiSep LSTM: A Machine Learning Algorithm for Early Detection of Septic Shock

**DOI:** 10.1038/s41598-019-51219-4

**Published:** 2019-10-22

**Authors:** Josef Fagerström, Magnus Bång, Daniel Wilhelms, Michelle S. Chew

**Affiliations:** 1 Department of Computer and Information Science, Linköping University, Linköping, 581 83 Sweden; 2 Division of Drug Research, Department of Medical and Health Sciences, Faculty of Health Sciences, Linköping University, Linköping, 581 83 Sweden; 3 Department of Emergency Medicine, Local Health Care Services in Central Östergötland, Region Östergötland, Linköping, 581 91 Sweden

**Keywords:** Computational science, Computer science, Software, Diagnosis

## Abstract

Sepsis is a major health concern with global estimates of 31.5 million cases per year. Case fatality rates are still unacceptably high, and early detection and treatment is vital since it significantly reduces mortality rates for this condition. Appropriately designed automated detection tools have the potential to reduce the morbidity and mortality of sepsis by providing early and accurate identification of patients who are at risk of developing sepsis. In this paper, we present “LiSep LSTM”; a Long Short-Term Memory neural network designed for early identification of septic shock. LSTM networks are typically well-suited for detecting long-term dependencies in time series data. LiSep LSTM was developed using the machine learning framework Keras with a Google TensorFlow back end. The model was trained with data from the Medical Information Mart for Intensive Care database which contains vital signs, laboratory data, and journal entries from approximately 59,000 ICU patients. We show that LiSep LSTM can outperform a less complex model, using the same features and targets, with an AUROC 0.8306 (95% confidence interval: 0.8236, 0.8376) and median offsets between prediction and septic shock onset up to 40 hours (interquartile range, 20 to 135 hours). Moreover, we discuss how our classifier performs at specific offsets before septic shock onset, and compare it with five state-of-the-art machine learning algorithms for early detection of sepsis.

## Introduction

Sepsis is a serious condition that has a history of being ill-defined and difficult to diagnose. It is only recently that clinical consensus definitions of sepsis have been developed, thus removing a major obstacle for conducting comparative studies on the morbidity and mortality of sepsis^1,2^. The most recent definition of sepsis, Sepsis-3, defines sepsis as a “life-threatening organ dysfunction caused by a dysregulated host response to an infection”^2^. In layman’s terms, this means that the immune system causes damage to its own body’s tissues while fighting an infection. Septic shock is a subset of sepsis where one can observe circulatory, cellular, and metabolic abnormalities which are associated with greater mortality rates than regular sepsis. Importantly, several studies have shown that early treatment of patients with sepsis greatly improves their chance of survival^2–7^.

The symptoms caused by sepsis and septic shock are associated with average in-hospital mortality rates of at least 10% and 40% respectively, and data suggests that it can rise as high as 30% for sepsis and 80% for septic shock^2,8^. In developed countries, sepsis occurs in about 2% of all hospitalisations, with more than 50% of patients with severe sepsis requiring intensive care. The number of new cases each year per 100,000 people in the developed world is between 150 and 240 for sepsis, 50 and 100 for severe sepsis, and roughly 11 for septic shock. It is estimated that in the United States there are somewhere between 500,000 to more than 1,000,000 cases of sepsis every year^1,8^. In 2011, sepsis accounted for more than $20 billion (5.2%) of all hospital costs in the US^2^. In addition, it seems to be the case that the number of sepsis cases is increasing at a quicker rate than the population, possibly due to an increased median age^1,2,9,10^. A study spanning two decades, from 1979 to 2000, reported an annual increase of sepsis cases of around 8.7%^11^. There is less data available for low and middle income countries, but since infectious diseases are much more common than in the developed world it is reasonable to assume that sepsis is at least as common in the developing world as in the developed^11^.

Traditional scoring systems such as APACHE (Acute Physiology, Age, Chronic Health Evaluation)^12^, SAPS (Simplified Acute Physiology Score)^13^, and SOFA (Sequential Organ Failure Assessment)^2^ inform clinicians about disease severity and may help to discriminate between survivors and non-survivors, but they are not tools for early detection of sepsis. In order to diagnose sepsis, physicians rely on highly manifest changes in a combination of clinical variables in response to the physiological insult caused by an infection. However, temporal dependencies and subtle physiological changes may provide an indication of imminent sepsis prior to the life-threatening organ dysfunction that is required for fulfilling the current diagnostic criteria for sepsis. Since it is not humanly possible to consider all these effects and findings for every single patient, automatic analysis tools have to be developed.

Various machine learning algorithms have been investigated for early detection of sepsis. Following is a brief summary of five notable examples of such algorithms. In 2015, Henry *et al*.^14^ developed the Targeted Real-time Early Warning Score (TREWScore) for early detection of septic shock by fitting a Cox proportional hazards model^15^ to data extracted from the Multiparameter Intelligent Monitoring in Intensive Care (MIMIC-II) database^16,17^. Calvert *et al*.^18^ used time series analysis on data from MIMIC-II to develop a system for early detection of sepsis called “InSight”. In 2017, Harutyunyan *et al*.^19^ developed a multitask Long Short-Term Memory (LSTM)^20^ neural network for early detection of a host of conditions, including sepsis, using the Medical Information Mart for Intensive Care (MIMIC-III) database^17,21^. The same year Kam and Kim^22^ created SepLSTM, an LSTM network based on the work of Calvert *et al*., that can perform early detection of sepsis with greater accuracy than InSight. Lastly, in 2019 Liu *et al*. used several machine learning models to predict a hypothesized “pre-shock” state, leading to improved performance in identifying patients who are likely to develop septic shock^23^. For our study, we used these five algorithms as a representation of the state-of-the-art in early detection of sepsis and to provide a more comprehensive context in which our algorithm can be placed.

The goal of the study was twofold: (1) to develop an improved algorithm for early detection of septic shock and (2) to compare it with state-of-the-art algorithms for early sepsis detection. These algorithms can primarily be improved by increasing the number of correct predictions as well as by providing these predictions earlier since early treatment is a key factor for patient survival^2–7^. To improve comparability, we replicated the TREWScore study^14^, employing the same input variables and target definitions but substituting the Cox proportional hazards model for an LSTM network.

## Results

The LiSep LSTM model was created by training an LSTM network that predicts whether or not a patient is going to develop septic shock during his or her stay in the hospital. During training, the model is fed patient data from the MIMIC-III database where each patient is marked as positive or negative if they developed septic shock during their admission or not, respectively. The set of input features used is the same as that derived by Henry *et al*.^14^ during the creation of TREWScore, and includes patient biometrics, vital parameters, and laboratory test results sorted by the closest whole hour after admission. We also used the same definition for septic shock as Henry *et al*., the specifics of which can be found in the Methods section.

For the model evaluation, the full data set was split into six equal parts. Six instances of our model were then trained, each using a different set of five of these parts as training data, reserving the last as test data. The main reason for this is to show how well the model generalizes to unseen data while removing any potential bias present in any single training data set. Once the models had finished training, they were used to generate predictions for their respective test data sets, thus generating six separate sets of predictions. These six sets were then used to compute the two performance metrics used to evaluate the model: the area under the receiver operating characteristic curve (AUROC), and the number of hours by which the model’s first positive prediction precedes the onset of septic shock, here referred to as hours before onset (HBO). We also calculated the AUROC for each of the 48 hours directly preceding the onset of septic shock to gain insight into how the reliability of the predictions change as the onset draws closer.

A summary of the evaluated performance of LiSep LSTM is presented in Table 1 along with the reported performance of the five state-of-the-art models. For each model, the test data AUROC and HBO median is shown, including 95% confidence intervals (CI) for the AUROC and the interquartile range (IQR) of the HBO when these are available. The results show that LiSep LSTM performs better than or on par with all models with respect to either AUROC or HBO, or both. SepLSTM and Liu *et al*.‘s pre-shock RNN both show an AUROC of 0.93, but perform worse in terms of HBO, where LiSep LSTM stands out. It is important to emphasize the difference in the comparison with TREWScore and the comparisons with the five state-of-the-art algorithms. Whereas LiSepLSTM and TREWScore only differ in terms of model choice and the use of an updated database, no special care has been taken to ensure any conformity with the five remaining algorithms. Thus, through the comparison with TREWScore one can see the impact of the choice of model and database, while the comparison with the other algorithms is mainly useful as a pure performance comparison.

**Table 1:** AUROC and HBO for LiSep LSTM and the five state-of-the-art models.

Model Number	AUROC (95% CI)	HBO Median (IQR)
LiSep LSTM	0.83 (0.82, 0.84)	48 (20.0, 135.0)
TREWScore	0.83 (0.81, 0.85)	28.2 (10.6, 94.2)
InSight	0.83 (0.80, 0.86)	<3* (N/A)
Multitask LSTM	0.85 (N/A)	N/A (N/A)
SepLSTM	0.93 (N/A)	<3* (N/A)
Liu *et al*. pre-shock RNN	0.93 (N/A)	7.0 (N/A)

*Measured from the first sustained SIRS event.

**Figure 1:**
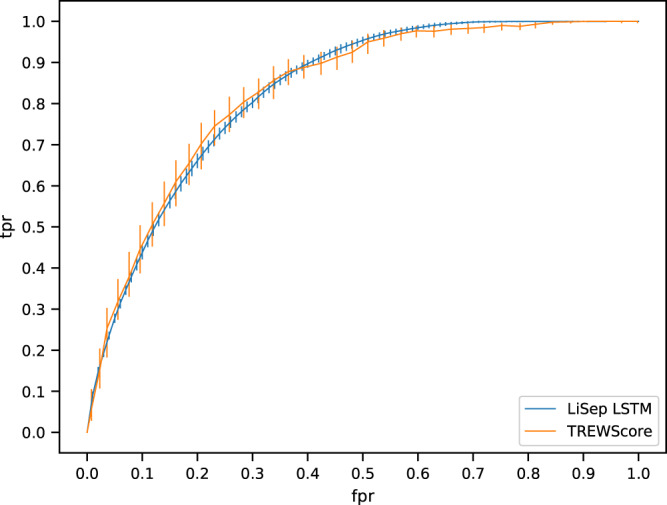
ROC curve for LiSep LSTM vs. TREWScore. Error bars show 95% CI. LiSep LSTM ROC curve (blue) was computed using test data. Confidence intervals were calculated by bootstraping the evaluation results for the six trained model instances. TREWScore ROC curve (orange) was extracted from the graph presented by Henry *et al*.

Figure 1 shows the test data ROC curve for LiSep LSTM as well as the ROC curve for TREWScore as presented by Henry *et al*.^14^. Since the AUROC is identical for both models (as can be seen in Table 1) it is not unexpected that the curves look very similar. The only real difference is that TREWScore has slightly larger confidence intervals.

**Figure 2:**
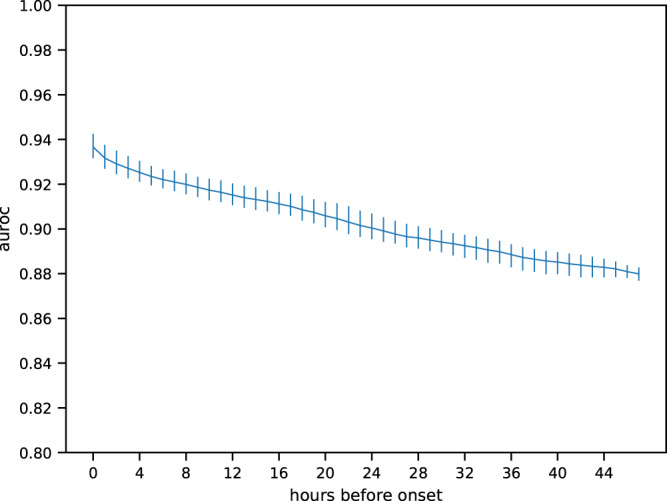
AUROC over the 48 hours directly preceding septic shock onset. Area under the ROC curve for LiSep LSTM (blue) when considering only predictions made within a number of hours before septic shock onset as indicated by the x-axis. Error bars show 95% CI and were generated by bootstrapping the evaluation results for the six trained model instances.

Figure 2 shows the AUROC for LiSep LSTM as it changes over the 48 hours directly preceding the onset of septic shock. As one might expect, the predictions become more reliable closer to the onset of septic shock. The implications of this are examined further in the Discussion.

## Discussion

In this study, we have demonstrated the benefit of using an LSTM network as opposed to the Cox proportional hazards model for early prediction of septic shock. Particularly interesting and of potential clinical importance is the significant increase in the HBO metric, since patients who are diagnosed early have a much higher chance of survival^2–7^.

Although LiSep LSTM shares similarities with all five algorithms previously mentioned, it is primarily an extension and replication of the research done by Henry *et al*.^14^. We used the same set of medical parameters as Henry *et al*. but substituted the Cox proportional hazards model for an LSTM network. Additionally, we used the MIMIC-III database which is an updated version of the database used for the development of TREWScore^17,21^. As a result, the comparison with TREWScore is the most interesting of the five models.

Interpreting the test data AUROC as a function of the time left to septic shock onset (see Fig. 2) is somewhat problematic. Although both Kim and Kam, as well as Henry *et al*. observe that their models perform better as sepsis or septic shock onset draws closer, they do not do any in-depth analysis or interpretation of this result. We argue that these results cannot be easily reduced to a simple performance measure, because it is not entirely clear what it means when the AUROC increases as septic shock onset draws closer. Certainly, for the test data it means that the model becomes more reliable as time passes, but this interpretation does not translate well to a real-life scenario since we do not know if and when a patient will develop septic shock until it has already happened. Therefore, additional information would be required in order to assess the reliability of a certain prediction. One possibility could be to create a model for estimating the time left until septic shock onset and combine the two models to yield a complete prediction adjusted for reliability. Either way, this problem requires further investigation.

The topology of the networks trained for this study was chosen in a somewhat unstructured manner based on an early exploratory analysis on the impact of each hyperparameter. The results presented in this paper indicate that the chosen parameter-values are at least not unreasonable, but it is likely that a more structured parameter selection process would yield an even better set of hyperparameters.

Two limitations of our performance analysis are related to the input features and the criteria used to determine whether or not a patient is septic shock positive. For this study, we decided to use the feature set developed by Henry *et al*. for TREWScore. This approach made the comparison between the two models more straightforward but the absolute performance of our model may have suffered as a result. As a comparison, SepLSTM used around 100 features compared to the roughly 30 we employed. Though the comparison is not completely valid since the prediction targets are different, it shows that there is significant variation in the possible valid feature sets and there may very well be set of features that is better suited for early prediction of septic shock. Additionally, deep learning methods, like LSTM networks, are known for being able to extract high-level features automatically without a separate feature extraction step. Unfortunately, the hardware available to us was too limited to allow us to explore these options in a timely fashion, and it had to be postponed to a future project. In a similar vein to the features, we had to use the now outdated sepsis definitions based on the Systemic Inflammatory Response Syndrome (SIRS) criteria to conform with Henry *et al*. Applying an outdated definition is appropriate for a comparative study like this one, but it might be beneficial or even necessary to retrain the model with an updated definition of sepsis if it is to be used in a clinical setting.

This study is also limited by the data set used for training and evaluation. Since the data in MIMIC-III is highly localised, being collected at a single hospital, it may be the case that the model cannot generalise well to patients in different places in the world. Thus, a logical study for the future would be a validation of this and other algorithms using MIMIC on other prospective data sets.

## Conclusion

In this study, we have showed that by using an LSTM network for early detection of septic shock, we can detect patients up to 20 hours earlier than a Cox proportional hazards model, at similar sensitivity and specificity, when the models are trained using the same features and target definitions. This finding is relevant since early detection and treatment of septic shock is essential for maximising the patient’s chance of survival.

## Methods

The data used for training and evaluation originates from the MIMIC-III database^17,21^. This database contains vital signs, laboratory tests, medical procedures, medications, journal notes, diagnoses, patient demographics, and mortality from approximately 59,000 admissions to the critical care units of Beth Israel Deaconess Medical Center between 2001 and 2012.

LiSep LSTM was created using the machine learning framework Keras^24^ with a Google TensorFlow^25^ back end. The model was trained on an NVIDIA GeForce GTX1080 Titan GPU with 11 GB memory. Some of the evaluation was done using an Intel i7-7700 CPU with 32 GB DDR4 RAM.

### Definition of septic shock

In order to retain algorithm comparability between LiSep LSTM and TREWScore, the definitions for sepsis, severe sepsis, and septic shock were based on the same criteria as Henry *et al*.^14^. In their study, sepsis was defined by the presence of any two of the SIRS criteria (shown below) in combination with the suspicion of an infection^26^:

- Body temperature: <36 °C or >38 °C
- Heart rate: >90 BPM
- Respiratory rate: >20 BPM or arterial CO_2_ pressure (PaCO_2_) < 32 mmHg
- White blood cell count: <4,000/mL or >12,000/mL
  Severe sepsis was defined as the presence of sepsis in combination with sepsis-related organ dysfunction. Sepsis-related organ dysfunction is specified in the surviving sepsis campaign guidelines as the presence of any of the symptoms listed below^27^:
- Systolic blood pressure: <90 mmHg
- Blood lactate: >2.0 mmol/L
- Urine output: <0.5 mL/kg over the last two hours despite adequate fluid resuscitation
- Creatinine: >2.0 mg/dL without the presence of chronic dialysis or renal insufficiency as indicated by ICD-9 codes V45.11 or 585.9
- Bilirubin: >2.0 mg/dL without the presence of chronic liver disease and cirrhosis as indicated by ICD-9 code 571 or any of its sub-codes
- Platelet count: <100,000/*μ*L
- International normalised ratio (INR): >1.5
- Acute lung injury with arterial O_2_ pressure (PaO_2_)/fraction of inspired oxygen (FiO_2_) < 200 in the presence of pneumonia as indicated by an ICD-9 code of 486
- Acute lung injury with PaO_2_/FiO_2_ < 250 in the absence of pneumonia

Septic shock was defined by the presence of severe sepsis and hypotension (systolic blood pressure <90 mmHg) despite adequate fluid resuscitation, defined as a fluid replacement over the past 24 hours ≥ 20 mL/kg or a total fluid replacement ≥1200 mL.

All patients were declared sepsis-negative or diagnosed with sepsis, severe sepsis, or septic shock on an hourly basis using these criteria. Patient and diagnosis statistics for our data set can be found in the Supplementary Materials.

When determining whether or not a patient should be considered septic shock-positive or negative Henry *et al*. considered an effect they called “censoring”. The reasoning was that when a patient receives treatment typical of that used to treat septic shock (e.g. fluid resuscitation), that specific patient’s condition gets censored in one of two ways. If the patient later fulfils the criteria for septic shock, the onset of the condition may have been delayed by the treatment. On the other hand, if the patient never fulfils the criteria for septic shock, the condition may have been pre-emptively treated. Henry *et al*. recognised that this might cause problems when fitting their model and they took special measures to deal with censored patients^14^. However, one of the benefits of LSTM networks is that they might be able to learn to recognise effects like censoring on their own, given that there are enough examples of it in the data set. Taking this into account, we decided not to explicitly consider censoring effects in our study.

### Feature extraction

The features, that is, the input variables used by our model were chosen to match, as closely as possible, the list in the Supplementary Materials of Henry *et al*.’s paper^14^. However, given that there are some differences between MIMIC-II and MIMIC-III, and that the Supplementary Materials in the paper do not entirely convey the feature extraction process used in the development of TREWScore, there are some differences between our data set and that used by Henry *et al*.

The feature extraction process began by filtering out all patients who were younger than 15 years old at the time of admission since the definition of sepsis is slightly different for children. The data was then subjected to a simple outlier detection step where values were removed if they fell outside the normal clinical range for that type of measurement. The specific feature ranges used are listed in Supplementary Table 3.

When dealing with missing values, Henry *et al*. first checked if a value had been recorded within a certain feature-specific time frame (e.g. 24 hours) prior to the time step with the missing value. If no such value existed, the population mean was used. Since we could not determine which specific time frames Henry *et al*. used, we opted for a simpler approach; after outliers had been removed, we used the Zero-Order-Hold (ZOH) method for dealing with missing values by imputing them using the most recent measurement of that feature for the patient and admission in question^28^. Features that had no previously recorded measurements for the current admission were replaced by the population mean^29,30^.

Finally, we normalised all non-binary features to a standard Gaussian curve using the formula $\hat{x}=(x-\bar{x})/s$ where *x* is the original value, $\hat{x}$ is the standardised value, $\bar{x}$ is the population mean, and *s* is the population standard deviation for the feature in question.

### Long short-term memory networks

**Figure 3:**
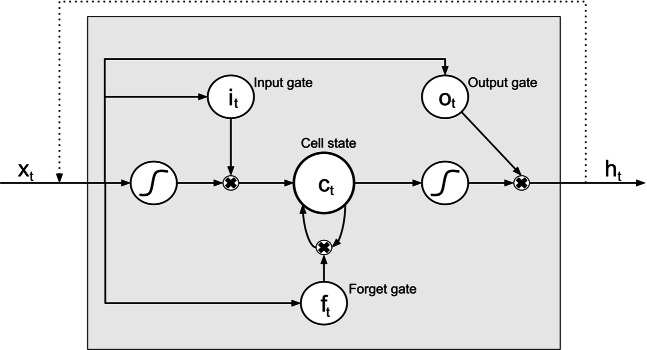
Overview of an LSTM neural processing unit. *x*_*t*_ is the input data, *h*_*t*_ is the hidden state, *i*_*t*_, *o*_*t*_, and *f*_*t*_ are gates controlling the flow of information, and *c*_*t*_ is the cell state. The S-shaped curves represent the application of an activation function. The black crosses represent the element-wise product.

LSTM networks is a subclass of Recurrent Neural Networks (RNN). In general, RNNs are used when dealing with data sequences of variable length, but in their basic form they are typically unable to learn to recognise long-term dependencies in the data. This problem was addressed by Hochreiter and Schmidhuber who in 1997 presented the LSTM node which can “learn to bridge minimal time lags in excess of 1,000 discrete-time steps”^20^. LSTM networks are constructed by combining several layers of LSTM units. Figure 3 shows the structure of an LSTM unit which consists of three gates that operate on the input vector, *x*_*t*_, to generate the cell state, *c*_*t*_, and the hidden state, *h*_*t*_. Intuitively, the cell state can be viewed as the cell’s “memory” while the gates control the flow of information in and out of the memory; the input gate determines how new information is incorporated, the forget gate determines which information to discard, and the output gate determines which information to pass along to the next layer.

The following set of formulas show how the hidden state for time t is calculated:

1 $$\begin{array}{rcl}{f}_{t} & = & {\sigma }_{g}({W}_{f}{x}_{t}+{U}_{f}{h}_{t-1}+{b}_{f})\\ {i}_{t} & = & {\sigma }_{g}({W}_{i}{x}_{t}+{U}_{i}{h}_{t-1}+{b}_{i})\\ {o}_{t} & = & {\sigma }_{g}({W}_{o}{x}_{t}+{U}_{o}{h}_{t-1}+{b}_{o})\\ {c}_{t} & = & {f}_{t}\circ {c}_{t-1}+{i}_{t}\circ {\sigma }_{c}({W}_{c}{x}_{t}+{U}_{c}{h}_{t-1}+{b}_{c})\\ {h}_{t} & = & {o}_{t}\circ {\sigma }_{h}({c}_{t})\end{array}$$

*W*_*q*_ and *U*_*q*_ are the weight matrices of the input and recurrent connections for the input gate, the output gate, the forget gate, or the cell state. *b*_*q*_ is the bias terms for the same components. $\circ$ is the element-wise product of two vectors. In this setup, *σ*_*g*_ is a sigmoid function, and *σ*_*c*_ and *σ*_*h*_ are hyperbolic tangent functions.

The network hyperparameters were chosen by hand based on early test runs which produced promising results. The final network consisted of four layers with 100 LSTM units each. A dropout probability of 0.4 was used and the network was trained for 1,000 epochs. In every epoch, 40 mini-batches, each consisting of 50 samples, where processed. Finally, since the number of negative cases is greater than the number of positive cases, we decided to increase the importance weighting for the positive cases to reduce this inherent bias of the dataset. We found that weighting the positive samples three times higher than the negative ones produced the desired effect. The factor three might seem low considering there are almost 10 times as many negative admissions as positive ones, but when adjusting for the varying length of stay we find that there are only roughly four times as many negative data points as there are positive ones.

Overfitting is a common issue when training any kind of neural network due to the highly flexible nature of such models. In order to reduce the negative effects of overfitting, one can apply regularisation techniques. One common regularisation technique for neural networks is *dropout*. When dropout is used during training, neurons and their connections are temporarily removed according to some predefined probability. This prevents the network from excessively adapting to the training data^31^. With some modifications this technique can successfully be applied to RNNs and LSTM networks^32^.

### Network evaluation

In order to obtain accurate performance measures of each network topology, we used six-fold cross-validation. We first set aside 5% of the total data to use as a validation set. The remaining 95% was split into six equal parts, or folds, five of which were used for training. The remaining fold was used as a test set after the training had finished to evaluate the performance of the network. This was repeated six times, each time using a different fold to evaluate the performance so that each fold was used as the test set exactly once. Moreover, we took special care to make sure that the proportion of negative to positive cases was roughly the same in all three data sets, as to reduce any negative effects stemming from extreme class imbalances.

The model was trained in two stages. During training, the AUROC was calculated for the training and validation data at regular intervals. Once the training of the model had finished, the ROC curve and its area, the HBO (time difference between the onset of septic shock and the model’s first positive prediction) for all correctly predicted septic shock-positive patients, and the AUROC for each of the 48 hours closest to septic shock onset were calculated for the test data. This evaluation was also done by Kam and Kim, although they limited the calculation to three hours before the first sustained SIRS event^22^. It is also congruent to the one made by Henry *et al*. where they calculated the number of septic shock-positive patients identified by TREWScore at each hour up to 120 hours before septic shock onset^14^. Although it makes a direct comparison with TREWScore difficult, we calculated the AUROC because it gives a more comprehensive view of the model’s performance. Once all six folds had been processed, the results were combined so the 95% CI and the IQR could be calculated for the ROC curve and the HBO respectively.

## Supplementary information

Supplementary Materials

## Data Availability

The raw evaluation data is publicly available at https://data.mendeley.com/datasets/gx9jcchdkk/1. For access to MIMIC-III, please refer to^21^ and^17^.
