# Supplementary material for: LiSep LSTM: A Machine Learning Algorithm for Early Detection of Septic Shock
Source: Sci Rep. 2019 Oct 22;9:15132. doi: 10.1038/s41598-019-51219-4 (PMC6805937; doi:10.1038/s41598-019-51219-4)
Supplement: Supplementary file 1 — Supplementary Materials [file 41598_2019_51219_MOESM1_ESM.pdf]

LiSep LSTM: A Machine Learning Algorithm for Early Detection of Septic Shock - Supplementary Materials

Fagerström, Josef,[1,*] Bång, Magnus[1,+], Wilhelms, Daniel[2,3,+] Chew, Michelle[2,+]

[1]Department of Computer and Information Science, Linköping University, Linköping, 581 83, Sweden
[2]Division of Drug Research, Department of Medical and Health Sciences, Faculty of Health Sciences, Linköping University, Linköping 581 83, Sweden
[3]Department of Emergency Medicine, Local Health Care Services in Central Östergötland, Region Östergötland, Linköping, 581 91, Sweden
[*]josef.fagerstrom@liu.se
[+]shared senior authorship

**Statistic**	**Amount**	%
Number of admissions	50373	100
Male patients	28304	56.2
Female patients	22069	43.8
Patients with sepsis	11224	22.3
Patients with severe sepsis	10115	20.1
Patients with septic shock	4786	9.50
Total IHD	5783	11.5
Sepsis IHD	2153	4.27 (19.2)
Severe sepsis IHD	2116	4.20 (20.9)
Septic shock IHD	1556	3.09 (32.5)
Unspecified 28-day mortality	1883	3.74
Sepsis 28-day mortality	715	1.42 (6.37)
Severe sepsis 28-day mortality	644	1.28 (6.37)
Septic shock 28-day mortality	302	0.60 (6.31)
Mechanical ventilation during ICU stay	14143	28.1

Supplementary Table 1: Population overview
IHD = In-hospital deaths. Number of patients who died in the hospital while exhibiting symptoms for the specified condition. Note that the specified condition is not necessarily the cause of death.
When two percentages are presented, the first one is relative to the total number of admissions, while the second one is relative to the number of patients afflicted by the condition indicated by the first column of the row.

**Unit**	**No. of admissions**	%
Coronary Care Unit (CCU)	7681	14.2
Cardiac Surgery Recovery Unit (CSRU)	9257	17.1
Medical Intensive Care Unit (MICU)	20926	38.8
Surgical Intensive Care Unit (SICU)	8841	16.4
Trauma Surgical Intensive Care Unit (TSICU)	6414	11.9
Unspecified	875	1.62

Supplementary Table 2: Number of admissions to each intensive care unit

**Feature name**	**Unit of measurement**	**Minimum**	**Maximum**
Age	-	15	90
Arterial pH	-	1	10
Diastolic Blood Pressure	mmHg	1	300
Fraction of Inspired Oxygen	%	5	100
Glasgow Coma Scale	points	0	15
Heart Rate	bpm	1	320
Mean Arterial Blood Pressure	mmHg	1	400
Platelet Count	K/μL	1	1500
Respiratory Rate	bpm	1	150
Riker Sedation-Agitation Scale	-	0	7
Systolic Blood Pressure	mmHg	1	400
Oxygen Saturation	%	1	100
Bicarbonate Level	mEq/L	1	50
Blood Urea Nitrogen	mg/dL	1	300
Creatinine	mg/dL	1	20
Hematocrit	%	1	100
Hemoglobin	g/dL	1	50
Potassium	mEq/L	0	20
Partial Carbon Dioxide Pressure	mmHg	1	70
Partial Oxygen Pressure	mmHg	1	200
White Blood Cell Count	K/μL	0.1	100
Fluid Input	mL	0	50,000
Antibiotics	-	-	-
Urine Output	mL	0	10,000

Supplementary Table 3: Ranges used for outlier detection
